# Oxysterols Profile in Zebrafish Embryos Exposed to Triclocarban and Propylparaben—A Preliminary Study

**DOI:** 10.3390/ijerph19031264

**Published:** 2022-01-24

**Authors:** Carmine Merola, Anton Vremere, Federico Fanti, Annamaria Iannetta, Giulia Caioni, Manuel Sergi, Dario Compagnone, Stefano Lorenzetti, Monia Perugini, Michele Amorena

**Affiliations:** 1 Faculty of Bioscience and Technology for Food, Agriculture and Environment, University of Teramo, 64100 Teramo, Italy; cmerola@unite.it (C.M.); avremere@unite.it (A.V.); ffanti@unite.it (F.F.); aiannetta@unite.it (A.I.); msergi@unite.it (M.S.); dcompagnone@unite.it (D.C.); mamorena@unite.it (M.A.); 2 Department of Food Safety, Nutrition and Veterinary Public Health, Istituto Superiore di Sanità—ISS, 00161 Rome, Italy; stefano.lorenzetti@iss.it; 3 Department of Life, Health and Environmental Sciences, University of L’Aquila, 67100 L’Aquila, Italy; giulia.caioni@guest.univaq.it

**Keywords:** oxysterols, propylparaben, triclocarban, zebrafish embryos, toxicity

## Abstract

Oxysterols have long been considered as simple by-products of cholesterol metabolism, but they are now fully designed as bioactive lipids that exert their multiple effects through their binding to several receptors, representing endogenous mediators potentially involved in several metabolic diseases. There is also a growing concern that metabolic disorders may be linked with exposure to endocrine-disrupting chemicals (EDCs). To date, there are no studies aimed to link EDCs exposure to oxysterols perturbation—neither in vivo nor in vitro studies. The present research aimed to evaluate the differences in oxysterols levels following exposure to two metabolism disrupting chemicals (propylparaben (PP) and triclocarban (TCC)) in the zebrafish model using liquid chromatography coupled with tandem mass spectrometry (LC-MS/MS). Following exposure to PP and TCC, there were no significant changes in total and individual oxysterols compared with the control group; however, some interesting differences were noticed: 24-OH was detected only in treated zebrafish embryos, as well as the concentrations of 27-OH, which followed a different distribution, with an increase in TCC treated embryos and a reduction in zebrafish embryos exposed to PP at 24 h post-fertilization (hpf). The results of the present study prompt the hypothesis that EDCs can modulate the oxysterol profile in the zebrafish model and that these variations could be potentially involved in the toxicity mechanism of these emerging contaminants.

## 1. Introduction

Over recent years, many environmental chemicals, including additives in the manufacture of plastic materials (e.g., bisphenol A (BPA)) and personal care products (e.g., parabens and triclocarban (TCC)), have been shown to possess the ability to interfere in hormone action [1]. Such endocrine-disrupting chemicals (EDCs) may act by interacting with all key regulatory steps of hormone systems, from hormone synthesis by the endocrine gland to the responses of hormone-responsive cells [2]. 

In addition to the developmental and reproductive effects, there is also a growing concern that metabolic disorders may be linked to EDCs [3]. Such EDCs have been defined as environmental obesogens [4]. However, because adverse effects by EDCs may also lead to other metabolic diseases such as nonalcoholic fatty liver disease (NAFLD), hyperlipidemia, and type 2 diabetes, this subclass would be better referred to as metabolic disruptors [3,4,5].

Metabolic disruption may result from different types of activity, including metabolic perturbations [3], interactions with specialized metabolic receptors [6] and xenosensors activation. These proteins are specialized in sensing the chemical environment and are typically involved in the activation of detoxification processes [7]. In addition, EDCs could influence metabolic homeostasis also through epigenetic modifications and mitochondrial dysfunction [8]. Due to the complexity of ED-associated mechanisms and pathways, the identification of molecular initiating events behind the metabolic phenotype effects of EDCs is a challenging task. 

Over the years, numerous endogenous mediators have been investigated to better understand metabolic syndromes. In this background, bioactive lipids such as bile acids, endocannabinoids, ceramides, and oxysterols seem to play a key role [9,10]. In particular, oxysterols gained importance, being considered more than by-products of cholesterol metabolism. They can derive from non-enzymatic processes, including cholesterol auto-oxidation mediated by reactive oxygen species, or enzymatic mechanisms, as intermediates in the formation of the bile acids and steroid hormones [9,10,11,12]. They are considered bioactive lipids, exerting their pleiotropic effects through multiple receptors such as nuclear receptors (LXRs, ERs, RORs, glucocorticoid receptors, GRs), G-protein-coupled receptors (GPCRs), and targeting also on regulatory proteins [13]. Variations in oxysterol levels have been reported in patients affected by metabolic syndromes [12], raising interest about the possibility of using oxysterols as markers of pathological conditions. For example, Samadi et al. (2018) reported that high levels of some types of oxysterols could positively correlate with coronary risks factors in patients affected by type 1 and 2 diabetes, giving information about oxidative stress [14]. In addition, Alkazemi et al. (2008) examined the relationship between the serum levels of oxysterols (7α-hydroxy-cholesterol, 7β-hydroxycholesterol, and 7-ketocholesterol) and the presence of metabolic alterations, reflected in obesity, insulin-resistance, and an increase in oxidative stress [15]. 

Since oxysterol levels reflect the status of metabolism, they are expected to be altered after exposure to environmental toxicants, and in this case, testing two well-known metabolic disrupters, propylparaben (PP) and triclocarban (TCC). They have recently emerged as chemicals able to disrupt yolk sac consumption and lipid metabolism in zebrafish early-life stages [16,17].

PP is the propyl ester of 4-hydroxybenzoic acid, and it is used as a preservative in personal care products and food. Its wide use is related to its antifungal and antimicrobial properties [18]. The concern about PP derives from its estrogenic properties and the evidence reported from several epidemiology studies. PP has been detected in human urines and matrices, such as breast milk, cord blood and placenta, seminal plasma, adipose tissue, and also breast cancer [19]. PP is an emerging contaminant, and its presence has been reported in wastewaters at concentration of 20,000 ng/L [20], in freshwater at 3142 ng/L, and also in bottled drinking water at 23 ng/L [21]. Negative effects on aquatic species have been also reported [22,23]. The detrimental effects mediated by PP are due to the ability to influence lipid metabolism [16], and in particular fatty acids metabolism [24], in line with its reported estrogen-like properties [25]. 

Triclocarban (TCC) is a diphenyl urea; it is an antimicrobial agent, which is added to many personal care products [26]. It is one of the contaminants of emerging concern, characterized by low solubility, which explains its tendency to persist in the environment (in surface waters, sediments) and to bioaccumulate in aquatic organisms [27]. It has been detected in human plasma, urine, umbilical cord blood, and milk, raising concerns about prenatal exposure in the developing fetus [28]. Influence on thyroid hormone function [29] and weak estrogenic effects [30] have been also reported. 

New and improved tools are needed to increase the quality, efficiency, and effectiveness of existing methods to evaluate the effects of metabolic disrupting chemicals, and zebrafish (*Danio rerio*) is increasingly used as an animal model to study the effects of EDCs, including those acting on metabolic homeostasis [31]. Due to its advantages (e.g., small size, short generation time, high fecundity, and rapid ex utero development of optically transparent embryos), different metabolic syndromes, such as hyperglycemia, obesity, diabetes, and hypertriglyceridemia, have been successfully studied using zebrafish [32,33,34]. Moreover, different oxysterols have been investigated in zebrafish as LX activators, and these mediators have also been related to neurodevelopment and immune function [35,36,37].

The present study aimed to evaluate the differences in oxysterols levels following exposure of zebrafish embryos to two different concentrations of PP and TCC. 22-Hydroxycholesterol (22-OH), 25-hydroxycholesterol (25-OH), 24-hydroxycholesterol (24-OH), 27-hydroxycholesterol (27-OH), 20d-hydroxycholesterol (20-OH), 7β-hydroxycholesterol (7b-OH), 7β-hydroxycholesterol-d7 (7b-OH-d7), and 7α-hydroxycholesterol (7a-OH) were detected in zebrafish embryos after exposure to these substances. 

## 2. Materials and Methods

### 2.1. Chemicals and Reagents

TCC (CAS number 101-20-02, Pharmaceutical Secondary Standard; Certified Reference Material), PP (CAS number 93-13-3, Pharmaceutical Secondary Standard; Certified Reference Material), and dimethyl sulfoxide (DMSO, >99.9% purity) were purchased from Merck Life Science (Milano, MI, Italia). 22-OH, 25-OH, 24-OH, 27-OH, 20-OH, 7b-OH, 7b-OH-d7, 7a-OH, and formic acid LC-MS grade were purchased from Sigma-Aldrich (Darmstadt, Germany). OMIX C18 cartridges from Agilent Technologies (Santa Clara, CA, USA); chloroform from Carlo Erba reagents (Milano, MI, Italy), methanol (MeOH), 2-propanol (ISOP), acetonitrile (ACN), and water, all UHPLC-MS grade solvents, from VWR (Radnor, PA, USA). Dilution water (DW) was prepared according to OECD TG 203, Annex 2 [38]; all salts (CaCl_2_, MgSO_4_, NaHCO_3_, KCl) were purchased from Sigma-Aldrich.

### 2.2. Zebrafish Maintenance and Eggs Collection

Adult zebrafish (wild-type AB strain) were bred in the University of Teramo facility (code 041TE294). Adults were kept in 3.5 L ZebTec tanks (Tecniplast S.p.a., Buguggiate, Italy) in a recirculating aquatic system. The temperature was maintained at 28 °C, pH at 7 ± 0.2, the conductivity at 500 ± 100 μS cm^−1^, and dissolved O_2_ at 6.1 mg L^−1^. The photoperiod was 14 h light–10 h dark. Chemical parameters were kept as follows: ammonia 0.02 mg L^−1^, nitrite 0.02 mg L^−1^, nitrate 21.3 mg L^−1^. Animals were fed twice a day with live food (*Artemia salina*) and supplemented with Zebrafeed 400–600 (Sparos, Olhão, Portugal).

The afternoon before spawning, ten groups of females and males (1:1) were introduced into 1.7 L breeding tanks (beach style design, Tecniplast S.p.a.). Immediately after spawning, which was initiated by morning light, fertilized eggs were collected with a sieve and rinsed thoroughly with deionized water and DW. Newly fertilized eggs were collected immediately after spawning and placed in groups of approximately 100 per Petri dish within a light- and temperature-controlled incubator until 2–3 hpf. Non-fertilized eggs and embryos with injuries were eliminated. 

### 2.3. Zebrafish Embryos Exposure

Stock solutions of each compound covering the tested concentration range were prepared in DMSO and stored at −20 °C. PP and TCC were tested at two concentrations: a toxicological concentration and an environmentally relevant concentration of human exposure. The toxicological concentrations were chosen to have effects on yolk sac resorption of zebrafish early-life stages—namely, TCC 50 µg/L and PP 1000 µg/L [16,17,18,19,20,21,22,23,24,25,26,27,28,29,30,31,32,33,34,35,36,37,38,39] —while the environmentally relevant concentrations included levels of exposure detected in the urine of pregnant women—namely, TCC 5 µg/L and PP µg/L [40]. Final concentrations of DMSO were 0.01% and 0.1% for TCC and PP, respectively. 

At 2–3 h post fertilization (hpf), embryos were examined under a dissecting microscope, and only the embryos that were developed normally and reached the blastula stage were selected for subsequent experiments. Afterward, embryos (4–16-cell stage) were transferred to glass beakers (diameter 115 mm, capacity 1000 mL) with 250 embryos in 400 mL of test solution. To prevent evaporation, glass Petri dishes were covered with self-adhesive transparent foil (SealPlate by EXCEL Scientific, Dunn, Asbach, Germany). Embryos were exposed for 24 hpf in the incubator at 27 ± 1 °C and photoperiod (14 h light–10 h dark) conditions. Oxysterol quantification was performed on viable embryos at 8–9 hpf (gastrula period) and 24 hpf (pharyngula period). Three replicates for each concentration were used. Prior to being used for the analytical procedure, zebrafish embryos were removed from the working solution with chemicals, washed two times with DW, and frozen at −80 °C. 

### 2.4. Quantification of Oxysterols Levels

For the quantitative analysis of oxysterols, zebrafish samples were analyzed with a Nexera LC20AD chromatographic system (Shimadzu, Kyoto, Japan) coupled with a Qtrap 4500 mass spectrometer (Sciex, Toronto, ON, Canada), and the analyses were performed according to Fanti et al. (2020) [41]. Briefly, the samples were sonicated in water and then vortexed; internal standard solution was added to 300 μL of sample to final concentration of 10 ng mL^−1^. The extraction was then performed in three steps by adding the extraction solvents, CHCl_3_, MeOH, and H_2_O, vortexing and mixing by means of an orbital shaker after each addition; finally, the mixture was centrifuged, and the CHCl_3_ portion was dried with a flow of N_2_, then resuspended in an ACN/MeOH/H_2_O solution. The extract was processed by micro Solid Phase Extraction (μSPE), using C18 OMIX tips, providing at the same time a suitable clean-up and a 4 time sample enrichment. 

The final extract was then analyzed by HPLC-MS/MS in multi reaction monitoring (MRM) acquisition mode, with electrospray ionization (ESI), providing a sensitive and accurate quantitation of the analytes, in addition to an unambiguous identification of the different isomeric forms.

### 2.5. Statistical Analysis

Data were assessed for normality by means of Shapiro–Wilk test. They were not normally distributed even after log and square root transformation, then Kruskal–Wallis test was applied. Differences were considered statistically significant at *p* < 0.05. SPSS^®^ 14.0.2 (SPSS Inc., Chicago, IL, USA) was used as the statistical package.

## 3. Results

**Figure 1:**
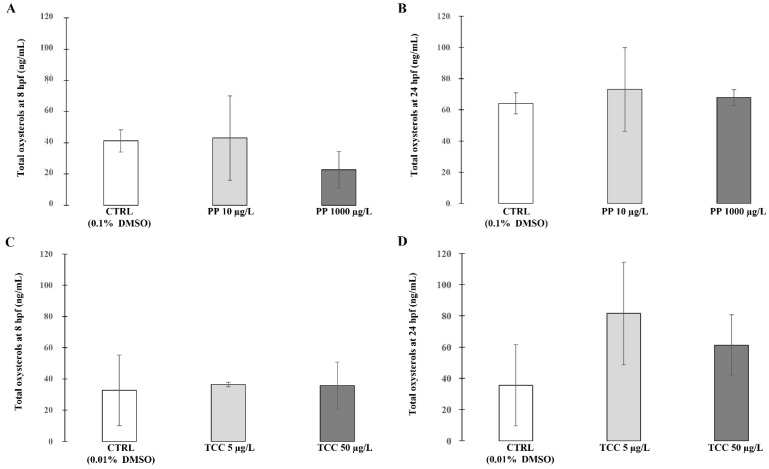
Total oxysterol concentrations in zebrafish embryos at 8 hpf and 24 hpf. The oxysterols concentrations are reported as ng/mL and represent the mean values of three independent measurements (±standard deviation).

Results of total oxysterol concentrations at 8 hpf and 24 hpf in the PP and TCC treated groups and in the control group are reported in Figure 1 and Table S1. Zebrafish embryos exposed to PP and TCC did not show any significant change in total oxysterols amounts in comparison with the CTRL groups. 

At 8 hpf, the oxysterols concentrations (mean value ± SD of 22.73 ± 11.80 ng/mL) in zebrafish embryos exposed to PP 1000 µg/L was lower compared with the CTRL group (41.32 ± 7.08).

**Figure 2:**
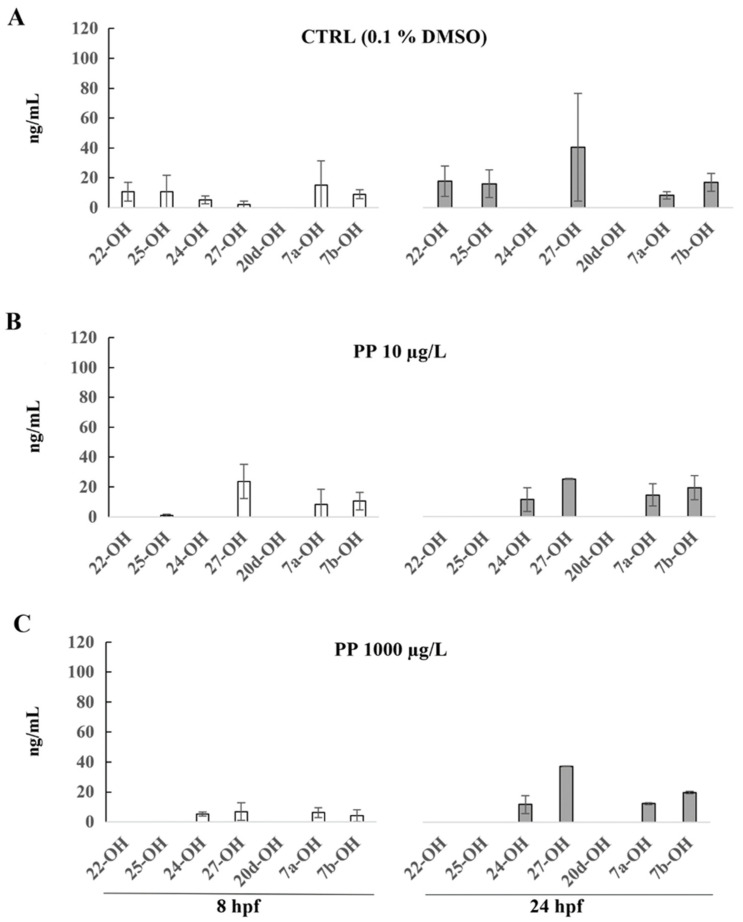
(**A**) Oxysterol profile in zebrafish embryos treated with 0.1% of DMSO (**A**), PP 10 µg/L (**B**)**,** and PP 1000 µg/L (**C**) at 8 hpf and 24 hpf. The oxysterols concentrations are reported as ng/mL and represent the mean values of three independent measurements (±standard deviation).

**Figure 3:**
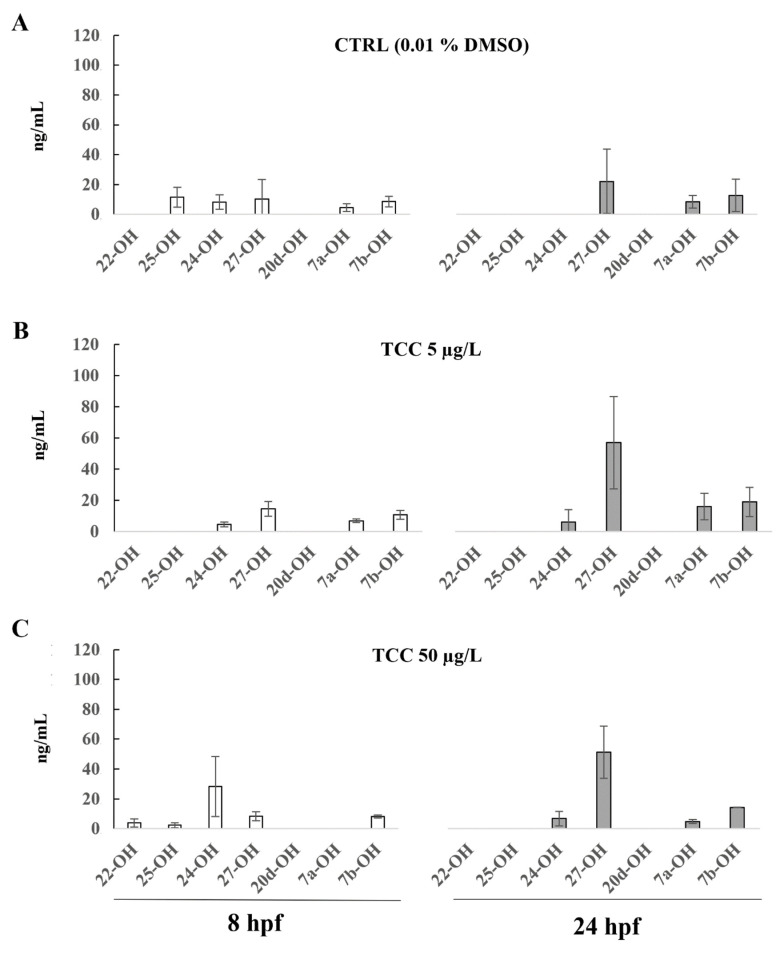
(**A**) Oxysterol profile in zebrafish embryos treated with 0.01% of DMSO (**A**), TCC 5 µg/L (**B**), and TCC 50 µg/L (**C**) at 8 hpf and 24 hpf. The oxysterols concentrations are reported as ng/mL and represent the mean values of three independent measurements (±standard deviation).

The PP and TCC pattern on the production of the individual oxysterols are shown in Figure 2 and Figure 3, and the mean values and SD are reported in Tables S2 and S3. The 20-OH was below the quantification limit at both time points and in both concentrations. 

In zebrafish embryos exposed to DMSO 0.1%, six different oxysterols (22-OH, 25-OH, 24-OH, 27-OH, 7a-OH, and 7b-OH) were detected at 8 hpf and 24 hpf. 

In PP treated embryos, the concentration of individual oxysterols did not show significant changes compared with the control group (Figure 2). 

At 8 hpf, 22-OH and 25-OH oxysterols were not detectable in zebrafish embryos exposed to both concentrations of PP. The levels of 7a-OH and 7b-OH were reduced and the concentrations of 27-OH increased in PP treated embryos compared with CTRL. 

At 24 hpf in zebrafish embryos exposed to PP, 22-OH and 25-OH oxysterols were not detectable, whereas the concentration of 24-OH oxysterol was higher compared with the CTRL. The levels of 7a-OH and 7b-OH oxysterols in 24 hpf PP treated embryos were similar to those observed in the CTRL group, except for the concentration of 27-OH oxysterol. 

Zebrafish embryos exposed to TCC did not show significant changes in individual oxysterols levels compared with CTRL (Figure 3). 

At 24 hpf, 27-OH showed an increase in TCC treated embryos compared with CTRL, and 24-OH was also detectable. The concentrations of 7a-OH and 7b-OH increased in zebrafish embryos exposed to TCC 5 µg/L compared with CTRL.

At 8 hpf in zebrafish embryos exposed to TCC 50 µg/L, the level of 24-OH was higher compared with CTRL group, and 22-OH was also detectable. In embryos exposed to TCC, the concentrations of 25-OH were lower compared with CTRL group, whereas 27-OH, 7a-OH, and 7b-OH increased at TCC 5 µg/L.

## 4. Discussion

Studies conducted on mouse models demonstrated that maternal exposure to realistic concentrations of TCC causes an increase in offspring body weight, alterations in lipid metabolism, and bioaccumulation of TCC-related compounds in organs, such as the brain, fat, muscles, and heart [42]. This hypothesis was also studied in the zebrafish model, in which TCC exposure induced an increase in neutral lipids content in the zebrafish larvae yolk [39]. The same alteration, in addition to a decrease in PLA2 activity, was also observed in zebrafish larvae treated with sublethal PP concentrations [16]. In agreement with these results, Bereketoglu and Pradhan (2019) showed that the apolipoprotein genes involved in fatty acid transport (apoab, apoeb, apoa4) and fatty acid synthesis (fasn) are downregulated in zebrafish larvae treated with PP [24]. The role of oxysterols as markers of toxicity of endocrine-disrupting chemicals is still unexplored.

Several EDCs can interact with CYP enzymes as well as being able to induce oxidative stress [43,44], leading to an increase and/or decrease in oxysterols concentrations in exposed organisms. PP and TCC are emerging endocrine disruptors with potential estrogenic activity [18,19,20,21,22,23,24,25,26,27,28,29,30,31,32,33,34,35,36,37,38,39,40,41,42,43,44,45], and as reported in the present study, the modulation of 27-OH concentrations following their exposure in the zebrafish model could represent an additional mechanism of estrogenic activity of these emerging contaminants. Estrogen receptors, on the other hand, are involved in insulin signaling and several metabolic processes [46]. Interestingly, the modulation of 27-OH depends on the tested chemical, on the concentration, and on the evaluation time. At 24 hpf, zebrafish embryos exposed to both concentrations of TCC showed an increased level of 27-OH oxysterols compared with the control group; on the contrary, no modifications happened in PP treated embryos. Plasma levels of 27-OH were increased in hypercholesterolemic patients and people with nonalcoholic fatty liver disease compared with healthy controls [47]. In the zebrafish model, 27-OH is synthesized by CYP27A1, and the expression of this enzyme starts from 24 hpf to 96 hpf [48]. The increased levels of 27-OH at 24 hpf compared with 8 hpf, both in treated and untreated zebrafish embryos, could be related to the activation of CYP27A1 enzymatic activity. 

Although the liver is the most studied organ responsible for oxysterol generation, they are also produced in other organs due to the expression of CYP in numerous tissues [9]. In the brain, the main cholesterol oxidation reaction, which is catalyzed by the brain-specific cholesterol 24-hydroxylase (CYP46A1), leads to the production of 24-OH exported from the brain to the systemic circulation [49]. 24-OH is a positive allosteric modulator of N-methyl-d-aspartate receptors (NMDARs) [50], and it is potentially involved in neurological disorders including Alzheimer’s disease, Smith–Lemli–Opitz Syndrome, Parkinson’s disease, multiple sclerosis, Huntington’s disease, amyotrophic lateral sclerosis, Niemann–Pick C disease, and autism spectrum disorders [47,48,49,50,51]. Interestingly, in treated embryos at 24 hpf, the levels of 24-OH were higher compared with the control group. Both parabens and triclocarban have shown neurotoxicity in in vitro and in vivo studies [52,53], and the potential role of 24-OH in EDCs neurotoxicity needs further investigations. 

25-OH, 7a-OH, and 7b-OH are produced both enzymatically through CH_25_H, CYP7A1, and 11bHSD1, respectively, and non-enzymatically through ROS production tissues [9]. 25-OH is a known ligand of the nuclear receptors LXRs, and despite it playing an important role in multiple metabolic pathways, it is also involved in the inflammation process [36]. In fact, the interferon-independent antiviral role of 25-OH was extended to a non-mammalian species, including teleost fish, where viral replication was also negatively affected by 25-OH administration to the zebrafish cell line ZF_4_ [37]. However, the toxicity of 25-OH was recently evaluated in the zebrafish model, where the exposure to this oxysterol impaired neuromuscular development, survival, and behavior, probably due to uncontrolled inflammatory responses in the treated organisms [36]. Following the obtained results, it could be possible to suppose that; PP exposure reduced the level of 25-OH both at 8 and 24 hpf, and this reduction could lead to an increased susceptibility to infections in treated embryos. 

7a-OH and 7b-OH were increased after exposure to 5 µg/L of TCC both at 8 and 24 hpf. The increased level of 7b-OH could be related to the production of ROS induced by TCC exposure. In fact, the enzyme (11bHSD1) responsible for the synthesis of this oxysterol is expressed starting from 120–144 hpf [48]. Moreover, the exposure of zebrafish embryos to TCC can activate oxidative stress, induce total antioxidant capacity expression and lipid peroxidation, and increase the activities of superoxide dismutase and other antioxidant enzymes to resist oxidative damage [54].

## 5. Conclusions

In conclusion, the present study demonstrated for the first time that exposure to such emerging endocrine disruptors as PP and TCC modulated the oxysterol concentrations in zebrafish embryos at 8 and 24 hpf. These results prompt the hypothesis that EDCs can modulate the oxysterol profile in the zebrafish model and that these variations could be potentially involved in the toxicity mechanism of these emerging contaminants.

The analytical method used for the determination of oxysterols could be applied also to study the effects of other endocrine disruptors on oxysterol profile of in vivo models. Further studies are needed to evaluate the consequences of oxysterols variations at biological levels and the potential role of these molecules as a toxicological biomarker.

## Data Availability

Not applicable.

## Supplementary Materials

The following are available online at https://www.mdpi.com/article/10.3390/ijerph19031264/s1, Table S1: Total oxysterols mean values ± SD at 8 and 24 hpf reported after the PP and TCC treatment. Table S2: Oxysterol mean values ± SD in zebrafish embryos treated with PP and TCC at 8 hpf. The oxysterols concentrations are reported as ng/mL. Table S3: Oxysterol mean values ± SD in zebrafish embryos treated with PP and TCC at 24 hpf. The oxysterols concentrations are reported as ng/mL.
