# Supplementary material for: Oxysterols Profile in Zebrafish Embryos Exposed to Triclocarban and Propylparaben—A Preliminary Study
Source: Int J Environ Res Public Health. 2022 Jan 24;19(3):1264. doi: 10.3390/ijerph19031264 (PMC8834710; doi:10.3390/ijerph19031264)
Supplement: Supplementary file 1 [file ijerph-19-01264-s001.zip › ijerph-1537806-supplementary.pdf]

Table S1. Total oxysterols mean values ± SD at 8 and 24 hpf reported after the PP and TCC treatment.

	**Mean ± SD at 8 hpf**	**Mean ± SD at 24 hpf**
DMSO 0.1%	41.32 ± 7.08	64.09 ± 6.82
PP 10 µg/L	43.08 ± 26.98	73.11 ± 26.76
PP 1 mg/L	22.73 ± 11.80	67.86 ± 5.18
DMSO 0.01%	32.68 ± 22.61	35.51 ± 26.12
TCC 5 µg/L	36.38 ± 1.48	81.60 ± 32.92
TCC 50 µg/L	35.76 ± 15.05	61.37 ± 19.37

Table S2. Oxysterol mean values ± SD in zebrafish embryos treated with PP and TCC at 8 hpf. The oxysterols concentrations are reported as ng/mL.

	**22-OH**	**25-OH**	**24-OH**	**27-OH**	**7a-OH**	**7b-OH**
DMSO 0.1%	10.77 ± 6.22	12.11 ± 11.2	5.18 ± 2.55	4.17 ± 2.41	13.04 ± 9.42	8.99 ± 3.11
PP 10 µg/L	<LOQ	1.03 ± 0.59	<LOQ	23.72 ± 15.94	9.31 ± 8.25	10.54 ± 5.92
PP 1 mg/L	<LOQ	<LOQ	5.33 ± 1.28	6.91 ± 5.93	6.25 ± 3.21	4.26 ± 3.95
DMSO 0.01%	<LOQ	11.54 ± 6.66	8.89 ± 4.08	15.31 ± 13.25	4.53 ± 2.51	8.61 ± 3.57
TCC 5 µg/L	<LOQ	<LOQ	4.45 ± 1.45	14.54 ± 4.76	6.78 ± 1.05	10.63 ± 2.88
TCC 50 µg/L	3.86 ± 2.74	2.39 ± 1.69	28.36 ± 20.05	8.32 ± 2.94	<LOQ	8.21 ± 1.01

Table S3. Oxysterol mean values ± SD in zebrafish embryos treated with PP and TCC at 24 hpf. The oxysterols concentrations are reported as ng/mL.

	**22-OH**	**25-OH**	**24-OH**	**27-OH**	**7a-OH**	**7b-OH**
DMSO 0.1%	14.80 ± 8.54	13.36 ± 7.71	<LOQ	50.57 ± 30.07	6.87 ± 2.15	14.09 ± 4.96
PP 10 µg/L	<LOQ	<LOQ	12.18 ± 9.1	28.43 ± 0.4	16.39 ± 8.27	21.86 ± 8.99
PP 1 mg/L	<LOQ	<LOQ	9.88 ± 5	31.16 ± 0.16	10.31 ± 0.6	16.52 ± 0.63
DMSO 0.01%	<LOQ	<LOQ	<LOQ	27.34 ± 17.93	6.84 ± 3.51	10.57 ± 9.09
TCC 5 µg/L	<LOQ	<LOQ	14.13 ± 6.64	47.38 ± 24.66	13.41 ± 7.06	15.76 ± 7.83
TCC 50 µg/L	<LOQ	<LOQ	5.62 ± 3.97	42.63 ± 14.57	4.02 ± 1.02	11.91 ± 0.19
